# Comment to: non-specific complaints at emergencydepartment presentation result in uncleardiagnoses and lengthened hospitalization: a prospective observational study

**DOI:** 10.1186/s13049-018-0553-7

**Published:** 2018-11-19

**Authors:** Roland Bingisser, Christian H Nickel

**Affiliations:** Department of Emergency Medicine, University Hospital Basel, Petersgraben 2, CH-4031 Basel, Switzerland

**Keywords:** Nonspecific complaints, Non-specific, Emergency department, Mortality, Geriatric emergency medicine

We would like to congratulate Sauer et al. for their study on the outcomes in patients presenting with nonspecific complaints (NSC) [1] and would like to comment on certain aspects of their publication.

First, the definition of NSC may be discussed controversially [2]. The authors used our original definition [3] and showed a near perfect inter-rater agreement with a kappa of 0.9 when retrospectively classifying symptoms to be specific or nonspecific. Therefore, post-hoc classification can be questioned to be truly prospective, as the data were obtained previously, and the raters were not blinded to outcomes or diagnoses. However, the authors showed impressively that this definition of NSC appears to be reliable and reproducible.

Second, the statement that “prospective comparisons of the outcomes of emergency patients with nonspecific complaints versus specific complaints are lacking” needs clarification. Taking a positive definition of NSC (on the basis suggested by Bhalla [4]), we have previously shown in a consecutive sample of 3960 ED visits [5] that “generalized weakness”, the most prevalent nonspecific symptom, [3] has serious outcomes; namely significantly increased hospitalization, ICU admission, in-hospital, and 1-year mortality, as compared to specific complaints. Therefore, direct previous prospective comparisons between NSC and other complaints have shown deleterious outcomes irrespective of the rule-out [3, 6] or rule-in [4, 7] definitions of NSC [5]. This is in agreement with previous retrospective studies as well [8, 9].

Third, the most important difference between the BANC cohort and Sauter’s cohort was the patient inclusion procedure. While Karakoumis [10] prospectively included 1300 patients presenting with NSC in a multi-centre setting, Sauter included 165 patients hospitalized in a single centre.

Taken together, in spite of major differences in the inclusion process and the definitions used, outcomes in patients with NSC from most studies are similar. Several prospective studies now prove this fact.
